# Reporting adverse events in a large community-based public health trial (building blocks): what gets reported and how does it vary?

**DOI:** 10.1186/1745-6215-14-S1-P135

**Published:** 2013-11-29

**Authors:** Gwenllian Moody, Eleri Owen-Jones, Rebecca Cannings-John, Carolyn Wallace, Mike Robling, Julia Sanders

**Affiliations:** 1 Cardiff University, Wales, UK; 2 University of South Wales, Wales, UK

## Background

Adverse event (AE) reporting is an integral part of safety monitoring for clinical trials and standard definitions for medical AEs exist under GCP. However, public health trials in community settings present challenges for consistent safety monitoring. For studies of complex behaviour change interventions, what should be recorded may not be self-evident. Building Blocks is such a trial and is evaluating the effectiveness of a home visiting intervention (Family Nurse Partnership programme). This presentation aims to assess variability in safety reporting, and explore factors associated with nature, level and quality of reporting.

## Methods

1,645 first-time mothers aged 19 or under were recruited across 18 sites. SAEs were reported during the 2.5 year follow-up period. All SAE forms were reviewed and categorised by an independent rater as either medical SAEs or AEs (standard GCP definition), or non-medical SAEs (using emergent study-specific definitions).

## Results

Over 1700 AE forms were returned. Reporting rates (reports per participants per site) varied considerably by site (22% to 465%). The results of the categorising exercise is to follow, and exploration of factors predictive of SAE reporting will be reported (e.g. site, participant, researcher).

## Conclusions

In all trials AEs may occur and require a robust monitoring process. The nature of non-medical AEs may be especially important in some trials. Researchers need to understand what may be relevant and ensure consistent and valid reporting. Such information will guide trial management (e.g. alert interviewers prior to follow-up assessments), and ensure harmful effects potentially attributable to the intervention are monitored.

